# Clinical pharmacology of cancer therapies in older adults

**DOI:** 10.1038/sj.bjc.6604201

**Published:** 2008-02-05

**Authors:** A Hurria, S M Lichtman

**Affiliations:** 1 The Department of Medical Oncology and Therapeutics Research, City of Hope, Duarte, CA 91010, USA; 2 Memorial Sloan-Kettering Cancer Center, Commack, NY 11725, USA

**Keywords:** pharmacokinetics, older patient, geriatric oncology, cancer therapy

## Abstract

This abbreviated review outlines the physiologic changes associated with aging, and examines how these changes may affect the pharmacokinetics and pharmacodynamics of anticancer therapies. We also provide an overview of studies that have been conducted evaluating the pharmacology of anticancer therapies in older adults, and issue a call for further research.

Cancer is a disease of older adults. Approximately 60% of cancer diagnoses and 70% of cancer mortalities occur in individuals age 65 and older (Yancik and Ries, 2000). As the general population ages and life expectancy increases, the number of older adults with cancer is growing.

Several unique challenges arise in caring for older adults with cancer. In particular, the physiologic changes associated with aging can have an impact on the pharmacokinetics and pharmacodynamics of cancer therapies. The effects of these age-related changes on drug dosing and tolerance have been understudied, as clinical trials that set the standards for oncology care and drug approval have typically focused on a younger patient population (Hutchins *et al*, 1999; Talarico *et al*, 2004). Few studies have included patients who are frail or who have a poor performance status (Table 1).

In this review, we provide an overview of the physiologic changes that accompany aging that may have an impact on the pharmacology of anticancer therapies. We also discuss recent studies evaluating the pharmacology of anticancer therapies in older adults.

## PHYSIOLOGIC CHANGES WITH AGING

Aging is a heterogenous process; however, some characteristic changes in physiology and organ function can have an impact on the pharmacology of anticancer therapy. For example, age-related changes in the gastrointestinal tract may affect drug absorption. These changes include a decrease in splanchnic blood flow, gastrointestinal motility, secretion of digestive enzymes, and mucosal atrophy (Yuen, 1990; Baker and Grochow, 1997). With increasing age, hepatic mass decreases and there is a decrease in the cytochrome p450 content in liver biopsies, although the impact of these declines on hepatic function remains controversial (Sotaniemi *et al*, 1997; Shah, 2004; Sawhney *et al*, 2005).

There is a decrease in renal mass and renal blood flow with aging (Vestal, 1997). These age-related changes in renal function could affect the pharmacology of anticancer drugs. A serum creatinine is often used to approximate renal function in younger adults; however, it is a poor indicator of renal function in older adults because of a decrease in muscle mass with age (Fehrman-Ekholm and Skeppholm, 2004). On average, the glomerular filtration rate decreases by approximately 0.75 ml min^−1^ year^−1^ after age 40; however, this decrease is not universal, and approximately one-third of all patients will have no change in creatinine clearance with age (Lindeman *et al*, 1985). Various equations have been used to estimate glomerular filtration rate, including the Cockcroft/Gault, Jeliffe, Wright, and MDRD (modification of diet in renal disease) formulas. The Cockcroft/Gault and Jeliffe formulas have primarily been validated in younger patients without renal disease (Burkhardt *et al*, 2002; Rimon *et al*, 2004). The Wright formula is more accurate than the Cockcroft/Gault formula in patients with a glomerular filtration rate of >50 (Marx *et al*, 2004). The MDRD formula is more accurate than other formulas in patients with chronic renal disease. This formula takes into account age, sex, ethnicity, serum creatinine, blood urea nitrogen, and albumin (Levey *et al*, 1999; Lichtman *et al*, 2007).

With increasing age, body composition changes, body fat increases and total body water decreases. This in turn increases the volume distribution of drugs that are lipid soluble and decreases the volume distribution of drugs that are water soluble. Hypoalbuminaemia and anaemia can lead to an increase in the volume distribution of drugs that are bound to albumin or haemoglobin, respectively. There is also an increase in bone marrow fat and a decrease in bone marrow reserve with age. This decrease in bone marrow reserve places older adults at increased risk for myelosuppressive complications from chemotherapy (Vestal, 1997).

Each of these physiologic changes that accompany aging could affect the pharmacokinetics and pharmacodynamics of anticancer therapies.

## CANCER THERAPY STUDIES IN OLDER ADULTS

Several recent studies have examined the effects of age-related changes on the pharmacokinetics and pharmacodynamics of common cancer therapies.

### Taxanes

Taxanes are primarily metabolised hepatically. The pharmacokinetics of paclitaxel 175 mg m^−2^ given every 3 weeks was evaluated in 153 patients, ages 55–86. With higher age, there was an increase in the area under the curve (AUC) of paclitaxel and a decrease in drug clearance. This translated into an age-related increase in grade 3 neutropenia and lower absolute neutrophil count (ANC) nadir; however, there was no increase in the incidence of fever, hospitalisation, or need for intravenous antibiotics (Lichtman *et al*, 2006). Common comorbid medical conditions in older adults may impact tolerance to cancer therapy. For example, patients with diabetic neuropathy may be at increased risk for neurotoxicity to taxanes (Rowinsky *et al*, 1993).

The pharmacokinetics of docetaxel at 75 mg m^−2^ every 3 weeks was evaluated in 40 patients. There was no significant difference in the pharmacokinetics of docetaxel in patients older or younger than age 65; however, older adults were more likely to experience febrile neutropenia and grade 4 neutropenia (ten Tije *et al*, 2005). A population pharmacokinetics study of 640 patients who received docetaxel demonstrated a small overall impact related to age, but a large impact of abnormal liver function tests on docetaxel clearance. On the basis of these data, the authors recommended that dose adjustments be made in patients with abnormal liver function tests; however, no specific dose adjustment was recommended based on age (Bruno *et al*, 2001).

Studies evaluating the pharmacokinetics and pharmacodynamics of weekly paclitaxel and docetaxel have yielded conflicting results (Fidias *et al*, 2001; Smorenburg *et al*, 2003; Minami *et al*, 2004; Slaviero *et al*, 2004; Hurria *et al*, 2006). The majority of these studies revealed no significant age-related differences in the pharmacokinetics of weekly taxanes; however, one small study (*N*=8) noted a 50% decrease in the clearance of unbound paclitaxel with increasing age (Smorenburg *et al*, 2003).

A newer formulation of taxane, nab-paclitaxel, is albumin-bound and Cremophor-free, eliminating the need for steroid premedication. This makes it an attractive option for older adults, particularly those with comorbidities such as diabetes, which can be exacerbated with steroid premedication (Nyman *et al*, 2005). To date, no studies have been conducted into age-related changes in the pharmacokinetics of nab-paclitaxel, and this offers an important area for future research.

### Alkylating agents

#### Cisplatin and carboplatin

Both cisplatin and carboplatin rely on renal clearance and have a similar mechanism of action. The clearance of the drugs is triphasic in nature, consisting of a distribution half-life (13 min for cisplatin; 22 min for carboplatin), elimination half-life (43 min for cisplatin; 116 min for carboplatin), and terminal half-life (5.4 days for cisplatin; 5.8 days for carboplatin). Potential cisplatin toxicities that require early evaluation, dose modification, and/or treatment in older adults include nephrotoxicity, nausea and vomiting, electrolyte abnormalities, ototoxicity, myelosuppression, and peripheral neuropathy. Cisplatin can be directly nephrotoxic to the renal tubules; therefore, it must be used with caution in older adults who may already have impaired renal function. The dose of cisplatin should be adjusted if the glomerular filtration rate is 30–50 ml min^−1^, and it should not be given if creatinine clearance is <30 ml min^−1^. The risk of cisplatin-induced nephrotoxicity can be ameliorated with intravenous hydration, and mannitol or furosemide diuresis. Carboplatin is not directly toxic to the renal tubules; however, since the drug is cleared renally, renal impairment will lead to increased drug levels. The clearance of carboplatin is linearly related to the patient's glomerular filtration rate. The Calvert formula, which takes into account a patient's glomerular filtration rate, is used to calculate the dose of carboplatin based on the desired AUC (Go and Adjei, 1999). A study of platinum-based chemotherapy for non-small cell lung cancer demonstrated that older patients were at increased risk for toxicity. In particular, compared with younger patients, older women were more likely to lose weight and older men were more likely to experience neuropsychiatric side effects and leucopenia (Langer *et al*, 2002).

#### Oxaliplatin

Oxaliplatin is a platinum compound that is increasingly being used for the treatment of colon cancer. The most common toxicities include myelosuppression, nausea/vomiting, and neuropathy. A pharmacokinetic study of oxaliplatin in 25 patients between the ages of 26 and 72 demonstrated no association between oxaliplatin pharmacokinetics and age; however, the clearance of oxaliplatin was associated with glomerular filtration rate (Graham *et al*, 2000). A pharmacokinetic study of oxaliplatin performed in 37 patients between the ages of 32 and 86, with varying degrees of renal dysfunction, and demonstrated an association between decreased creatinine clearance and decrease in the clearance of ultra-filterable platinum; however, there was no significant increase in side effects in patients with mild to moderate renal dysfunction. Therefore, the authors concluded that dose reductions were unnecessary in patients with a creatinine clearance of ⩾20 ml min^−1^ (Takimoto *et al*, 2003). The impact of severe renal impairment on the clearance of oxaliplatin still remains unknown. There is no significant alteration in the clearance of oxaliplatin in patients with liver dysfunction (Doroshow *et al*, 2003).

#### Temozolomide

Temozolomide is administered orally, and food decreases its absorption. The impact of age on the pharmacokinetics of temozolomide was evaluated in a cohort of 445 patients with a mean age of 50 (range 18–82) who were enrolled in phase I and II studies. There was no significant association between patient age and the pharmacokinetics of temozolomide; however, there was an association of age, female gender, and temozolomide exposure with myelosuppression. The most important factor influencing the clearance of temozolomide was body surface area: increased body surface area was associated with increased clearance (Jen *et al*, 2000).

### Vinca alkaloids

#### Vinorelbine

The vinca alkaloid, vinorelbine, is primarily excreted through the biliary tract. Dose adjustment in patients with severe biliary disease has been recommended. Two studies of small sample size evaluated the impact of age on the pharmacokinetics of vinorelbine, and yielded conflicting results. Sorio *et al* (1997) studied the pharmacokinetics of vinorelbine in 10 patients age ⩾65 and found no age-related change in pharmacokinetics or toxicity. In contrast, Gauvin *et al* (2000) evaluated the pharmacokinetics of vinorelbine in 12 patients age ⩾65 and found a significant age-related decrease in vinorelbine clearance, estimated as a 30–40% clearance decrease for patients age ⩾70. An increase in the AUC of vinorelbine was associated with an increased risk of haematologic toxicity (Gauvin *et al*, 2000). Further studies regarding the impact of age on the pharmacokinetics and pharmacodynamics of vinorelbine are needed.

### Antimetabolites

#### 5-fluorouracil

The antimetabolite, 5-fluorouracil (5FU), is metabolised hepatically. Its pharmacokinetics was evaluated in a cohort of 380 patients ages 25–91, but no significant impact of age was found. However, there were differences in 5FU clearance by gender. In particular, women had a significantly lower clearance of 5FU because of lower dihydropyrimidine dehydrogenase activity, the key enzyme involved in the clearance of 5FU (Milano *et al*, 1992). Other studies have demonstrated that older patients receiving 5FU are at increased risk for leucopenia and mucositis (Popescu *et al*, 1999; Sargent *et al*, 2001).

#### Capecitabine

The oral fluoropyrimidine, capecitabine, is absorbed in the gastrointestinal tract, then enzymatically converted to 5FU in the liver. Caution must be used in patients with liver dysfunction, as there are no clear dosing guidelines for capecitabine in patients with severe hepatic impairment. Capecitabine and its metabolites are primarily excreted in the urine, and a pharmacokinetics study of capecitabine in patients with varying degrees of renal dysfunction demonstrated that they have increased systemic exposure to capecitabine and an increased risk of grade 3 or 4 toxicity (Poole *et al*, 2002). The dose of capecitabine needs to be adjusted if a patient's creatinine clearance is <50 ml min^−1^, and capecitabine should not be prescribed in patients with a creatinine clearance <30 ml min^−1^.

There is a potential for drug interactions with capecitabine. Concomitant exposure to capecitabine and warfarin leads to exaggerated anticoagulant activity (Camidge *et al*, 2005). Thus, the international normalised ratio needs to be monitored carefully when prescribing capecitabine to a patient who takes warfarin. In addition, there is a potential for interaction between folate supplementation and capecitabine. In a study of patients with colorectal cancer receiving capecitabine, those with higher pretreatment levels of serum folate experienced greater toxicity during cycle 1 and over the treatment period (Sharma *et al*, 2006).

#### Methotrexate

Methotrexate, a component of the CMF (cyclophosphamide, methotrexate, 5FU) regimen for treatment of breast cancer, is associated with an increased risk of toxicity in older adults. This antifolate is eliminated renally and the clearance of methotrexate decreases with decreased creatinine clearance; therefore, the dosing of methotrexate should be adjusted based on renal function (Bressolle *et al*, 1998). In a study by Gelman and Taylor (1984), the age-related increase in toxicity with CMF was eliminated by modifying the doses of methotrexate and cyclophosphamide based on renal function, and empirically decreasing the dose of 5FU by two-thirds. However, it is not known if this approach affects the efficacy of the therapy (Gelman and Taylor, 1984). Caution is required in prescribing methotrexate for patients with metastatic cancer who have ascites or pleural effusion, which can lead to prolonged elimination of methotrexate and increased toxicity.

#### Cytosine arabinoside

Cytosine arabinoside (ara-C) is rapidly eliminated from plasma by deamination. Approximately 70–80% of a given dose is excreted as ara-U. Older adults are at increased risk for toxicity with high-dose ara-C because of age-related alterations in renal function. In particular, risk factors for ara-C-associated neurotoxicity include a creatinine ⩾1.2 mg dl^−1^, age ⩾40, and alkaline phosphatase ⩾3 times normal (Rubin *et al*, 1992). The incidence of neurotoxicity can be reduced if dosing takes renal function into account (Smith *et al*, 1997).

### Topoisomerase inhibitors

#### Etoposide

Etoposide is a topoisomerase II inhibitor. The clearance of etoposide is slower in patients with impaired renal function (Toffoli *et al*, 2001). In contrast, there is no significant difference in the pharmacokinetics of etoposide in patients with impaired hepatic function (Aita *et al*, 1999; Toffoli *et al*, 2001). Studies evaluating the impact of age on the pharmacokinetics of etoposide have produced conflicting results. Toffoli *et al* (2001) studied 50 patients ranging in age from 50 to 83. They found no impact of age on the pharmacokinetics of etoposide when taking creatinine clearance into account. Miller *et al* (1997) evaluated 106 patients who received etoposide in combination with cisplatin, and reported that older age was associated with an increase in free and trough etoposide concentrations. Ando *et al* (1999) evaluated the pharmacokinetics of oral etoposide in 12 patients ranging from age 75 to 84, and detected no difference in the etoposide pharmacokinetics of this group in comparison with historical data from younger patients. However, they did find that older patients were at increased risk for myelosuppressive complications.

### Anthracyclines

#### Doxorubicin

Doxorubicin is primarily metabolised hepatically. Li and Gwilt (2003) evaluated the impact of age on the pharmacokinetics of doxorubicin in 56 patients between the ages of 12 and 74. Increased age was associated with decreased clearance of doxorubicin. Dees *et al* (2000) evaluated the pharmacokinetics of AC (doxorubicin 60 mg m^−2^ and cyclophosphamide 600 mg m^−2^) in 24 patients. There was no impact of age on the clearance of doxorubicin or cyclophosphamide; however, older adults were at increased risk for myelosuppression. Age is a known risk factor for congestive heart failure in older adults who receive doxorubicin. Swain *et al* (2003) evaluated a cohort of 630 patients who received doxorubicin *vs* placebo in three phase III clinical trials, and found an association between older age and the risk of congestive heart failure after a cumulative doxorubicin dose of 400 mg m^−2^.

## FURTHER RESEARCH

There are limited data available regarding the impact of age on the pharmacokinetics and pharmacodynamics of targeted therapies. This area needs much more research. Some data do suggest an association between age and risk of toxicity with targeted therapies. For example, increasing age is a risk factor for congestive heart failure associated with trastuzumab, the humanised monoclonal antibody that targets the HER-2 (human epidermal growth factor receptor 2) protein. This was demonstrated in the NSABP B31 study of doxorubicin and cyclophosphamide followed by paclitaxel with or without trastuzumab for the adjuvant treatment of patients with HER-2/*neu*-positive breast cancer. In this study, risk factors for trastuzumab-associated congestive heart failure included older age (defined as age ⩾50) and a lower left ventricular ejection (defined as post-AC ejection fraction of 50–54%). There was a 20% (95% CI, 11.1–35.9), 3-year cumulative incidence of congestive heart failure in patients age ⩾50 who received adjuvant trastuzumab with a left ventricular ejection fraction of 50–54% after AC (Tan-Chiu *et al*, 2005). It is unclear whether this was solely a pharmacodynamic effect of trastuzumab on cardiac function or whether an age-related change in trastuzumab pharmacokinetics may also have played a role.

Another example of age-related toxicity with targeted therapy is seen among patients receiving bevacizumab, the humanised monoclonal antibody that inhibits vascular endothelial growth factor (VEGF). A retrospective pooled analysis of five randomised studies in 1745 patients demonstrated that patients age ⩾65, who were treated with chemotherapy and bevacizumab, had an increased risk of arterial thromboembolic events compared with those age <65 (7.1 *vs* 2.5%). Risk factors for development of an arterial thromboembolic event included age >65 (*P*=0.01) and history of a prior arterial thromboembolic event (*P*<0.001) (Scappaticci *et al*, 2007).

A phase III study (E3200) in patients with metastatic colon cancer receiving bevacizumab and 5FU, leucovorin, and oxaliplatin demonstrated that patients age ⩾65 had an increased risk of gastrointestinal side effects and fatigue compared with younger individuals. In addition, in a phase III study (E4599) of patients with advanced non-small cell lung cancer who received paclitaxel and carboplatin, and who were randomised to receive or not to receive bevacizumab, demonstrated that older adults were at increased risk for developing proteinuria.

There are limited data regarding the age-related pharmacokinetics or pharmacodynamics of other commonly used targeted agents, such as erlotinib, cetuximab, and sunitinib. A phase II study of erlotinib as first line therapy for patients age 70 and older with advanced non-small cell lung cancer demonstrated that 12% of patients on the study required discontinuation of therapy, most commonly due to interstitial lung disease, dehydration, and diarrhoea. There was one treatment related death attributed to interstitial lung disease. This trial did not include a direct comparison of older and younger adults; however, the rate of treatment discontinuation reported in this trial was higher than the rate of treatment discontinuation reported in previous clinical trials of patients with a younger mean age (Jackman *et al*, 2007).

The mechanism behind this age-related increase in side effects with targeted therapies is unclear, and studies are needed to address these questions.

## CONCLUSIONS

Despite the aging of the population and the association of cancer with aging, the pharmacokinetics and pharmacodynamics of anticancer therapies have been understudied in older adults. Most of the studies performed to date have not demonstrated significant age-related differences in the pharmacokinetics of cancer therapies; however, the majority of the studies consisted of small sample size and would not be able to detect a subtle change. In addition, chronologic age is only one factor to consider. Other measures of physiological age that may affect tolerance to cancer therapy in older adults should also be captured, including functional status, other comorbid medical conditions, concomitant medications, nutritional status, and renal and hepatic function. As new drugs are introduced into oncology treatment, phase II studies of the pharmacokinetics and pharmacodynamics of cancer therapies in older adults, which captures all of these factors, are needed in order to determine the optimum treatment for this growing population of older patients with cancer.

## Figures and Tables

**Table 1:** Impact of age on the pharmacokinetic (pK) studies of chemotherapy drugs

**Author**	**Drug**	**Ages**	**pK analysis (*n*)**	**Results**
Lichtman *et al* (2006)	Paclitaxel	55–86	153	Age-related decrease in clearance
Fidias *et al* (2001)	Paclitaxel	70–85	13	No age-related difference in pK
Smorenburg *et al* (2003)	Paclitaxel	22–84	23	Age-related decrease in clearance
Slaviero *et al* (2004)	Docetaxel	40–83	54	No age-related difference in pK
Hurria *et al* (2006)	Docetaxel	66–84	19	No age-related difference in pK
Minami *et al* 2004	Docetaxel and cisplatin	39–86	52	No age-related difference in pK
ten Tije *et al* (2005)	Docetaxel	26–80	40	No age-related difference in pK
Graham *et al* (2000)	Oxaliplatin	26–72	25	No age-related difference in pK
Jen *et al* (2000)	Temozolomide	18–82	445	No age-related difference in pK
Sorio *et al* (1997)	Vinorelbine	66–81	10	No age-related difference in pK
Gauvin *et al* (2000)	Vinorelbine	65–79	12	Age-related decrease in clearance
Milano *et al* (1992)	5-Fluorouracil	25–91	380	No age-related difference in pK
Cassidy *et al* (1999)	Capecitabine	41–80	25	No age-related difference in pK
Toffoli *et al* (2001)	Etoposide	50–83	50	No age-related difference in pK
Miller *et al* (1997)	Etoposide and cisplatin	<50–70+^a^	106	Age-related decrease in clearance
Ando *et alet al* (1999)	Etoposide	75–84	12	No age-related difference in pK
Li and Gwilt (2003)	Doxorubicin	12–74	56	Age-related decrease in Clearance
Dees *et al* (2000)	Doxorubicin and cyclophosphamide	a	24	No age-related difference in pK

a Exact age range not specified.
